# Neuroprotective Effects of a Traditional Multi-Herbal Medicine Kyung-Ok-Ko in an Animal Model of Parkinson's Disease: Inhibition of MAPKs and NF-κB Pathways and Activation of Keap1-Nrf2 Pathway

**DOI:** 10.3389/fphar.2018.01444

**Published:** 2018-12-13

**Authors:** Jong Hee Choi, Minhee Jang, Joon-Il Lee, Won-Seok Chung, Ik-Hyun Cho

**Affiliations:** ^1^Department of Science in Korean Medicine and Brain Korea 21 Plus Program, Graduate School, Kyung Hee University, Seoul, South Korea; ^2^Department of Convergence Medical Science, College of Korean Medicine, Kyung Hee University, Seoul, South Korea; ^3^Department of Korean Rehabilitation Medicine, College of Korean Medicine, Kyung Hee University, Seoul, South Korea; ^4^Institute of Korean Medicine, College of Korean Medicine, Kyung Hee University, Seoul, South Korea

**Keywords:** 1-methyl-4-phenyl-1, 2, 3, 6-tetrahydropyridine, Parkinson's disease, Kyung-Ok-Ko, anti-inflammation, anti-oxidation

## Abstract

Kyung-Ok-Ko (KOK), a traditional multi-herbal medicine, has been widely used in Oriental medicine as a restorative that can enforce vitality of whole organs and as a medicine that can treat age-related symptoms including lack of vigor and weakened immunity. However, the beneficial effect of KOK on neurological diseases such as Parkinson's diseases (PD) is largely unknown. Thus, the objective of this study was to examine the protective effect of KOK on neurotoxicity in 1-methyl-4-phenyl-1,2,3,6-tetrahydropyridine (MPTP)-induced mouse model of PD. Pre-treatment with KOK at 1 or 2 g/kg/day (p.o.) showed significant mitigating effects on neurological dysfunction (motor and welfare) based on pole, rotarod, and nest building tests. It also showed effects on survival rate. These positive effects of KOK were related to inhibition of loss of tyrosine hydroxylase–positive neurons, reduction of MitoSOX activity, increased apoptotic cells, microglia activation, and upregulation of inflammatory factors [interleukin (IL)-1β, IL-6, cyclooxygenase-2, and inducible nitric oxide], and reduced blood-brain barrier (BBB) disruption in the substantia nigra pars compacta (SNpc) and/or striatum after MPTP intoxication. Interestingly, these effects of KOK against MPTP neurotoxicity were associated with inhibition of phosphorylation of mitogen-activated protein kinases and nuclear factor-kappa B signaling pathways along with up-regulation of nuclear factor erythroid 2-related factor 2 pathways in SNpc and/or striatum. Collectively, our findings suggest that KOK might be able to mitigate neurotoxicity in MPTP-induced mouse model of PD via multi-effects, including anti-neuronal and anti-BBB disruption activities through its anti-inflammatory and anti-oxidative activities. Therefore, KOK might have potential for preventing and/or treating PD.

## Introduction

Parkinson's disease (PD) is the most common chronic neurodegenerative movement disorder characterized by profound loss of dopaminergic neurons, accumulation of α-synuclein (α-syn), and microglial and astroglial activation in the substantia nigra pars compacta (SNpc), leading to depletion of dopamine in the striatum (Shulman et al., 2011; Kalia and Lang, 2016; Poewe et al., 2017). Its key symptoms are slow movement, tremor, rigidity, and problems in cognition, memory, and emotion (Shulman et al., 2011; Kalia and Lang, 2016; Poewe et al., 2017). Current therapeutic strategies including levodopa only provide temporary relief for motor symptoms (Marsden, 1994; Athauda and Foltynie, 2015) without preventing loss of dopaminergic neurons. They may produce side effects including dizziness, nausea, and vomiting during prolonged treatment (Marsden, 1994; Athauda and Foltynie, 2015). These unsatisfactory effects of current therapeutic strategies (directed at a single target) might be related to the complex or multifactorial etiology of PD, including apoptosis, glutamate excitotoxicity, proteasomal dysfunction, mitochondrial dysfunction, oxidative stress, and environmental exposures (Riess and Krüger, 1999; Shulman et al., 2011; Kalia and Lang, 2016; Poewe et al., 2017).

To overcome limitations of drugs directed at a single target, a new paradigm of drug development (multi-targeted drugs) has been recently suggested based on highly complex and multi-factorial etiologies of various neurodegenerative disorders (Bajda et al., 2011; Dias and Viegas, 2014; Zheng et al., 2014; Calabresi and Di Filippo, 2015). Traditional multi-herbal medicines usually comprising multiple herbs in a single formula have been widely prescribed for a wide range of diseases and conditions (Newman and Cragg, 2016). These medicines can exert clinical effects with multi-target activities through chemical, pharmacological, and pharmaceutical functions of multi-herbs (Zeng, 2017).

Kyung-Ok-Ko (KOK; Qiong-yu-gao in Chinese; Kei-gyoku-kou in Japanese), a traditional multi-herbal medicine used for health improvement, is a decoction of six ingredients: *Rehmannia glutinosa Liboschitz* var. *purpurae* Makino (Scrophulariaceae), *Lycium chinense* Miller (Solanaceae), *Aquillaria agallocha* Roxburgh (Thymelaeaceae), *Poria cocos* Wolf (Polyporaceae), *Panax ginseng* C.A. Meyer (Araliaceae), and honey (Jang et al., 2014; Lee et al., 2016b). In Oriental countries, KOK has been prescribed as a restorative to enforce the vitality of the body or as a medicine to prevent or treat various age-related symptoms including lack of vigor and weakened immunity, emaciation, and cognitive impairment (Huh, 1610). Cumulating scientific evidences have demonstrated that KOK has cognitive-enhancing (Shin et al., 2009), anti-ischemic (Cai et al., 2011), anti-tyrosinase (Ye et al., 2010), and anti-cancer (Lee et al., 2002) effects. Additionally, we have recently demonstrated that KOK can inhibit the expression of macrophage, T cell, and inflammatory mediators in retroperitoneal lymph nodes, ovaries, and uteri of dehydroepiandrosterone (DHEA)-induced polycystic ovary syndrome (PCOS) and uterine abnormality model in rats (Jang et al., 2014; Lee et al., 2016b). These results suggest that KOK might exert positive effects against neurological disorders. However, little is known on the effect of KOK on PD. Therefore, the objective of this study is to examine the protective effect of KOK on neurotoxicity in 1-methyl-4-phenyl-1,2,3,6-tetrahydropyridine (MPTP)-induced mouse model of PD. Here we demonstrated that pre-treatment of KOK can inhibit neurological impairments and neuronal loss in SNpc and striatum in MPTP-induced neurotoxicity model in rat. Such effects of KOK are related to its multiple functions, including anti-neuronal death, anti-inflammatory, and anti-oxidant activities. These results suggest that KOK might have potential to prevent or treat PD.

## Materials and Methods

### Animals and Ethical Approval

Adult male C57BL/6 mice (Narabiotec Co., Ltd., Seoul, Republic of Korea) that were 7–8 weeks of age and weighed 21.5–22.5 g) were housed at a constant temperature of 23 ± 2°C with a 12-h light-dark cycle (lights on from 08:00 to 20:00), and provided with food and water *ad libitum*. All experimental procedures were reviewed and approved by the Institutional Animal Care and Use Committee and Institutional Biosafety Committee of Kyung Hee University. In this process, proper randomization of laboratory animals and handling of data were performed in a blinded manner in accordance with recent recommendations from an NIH workshop on preclinical models of neurological diseases (Landis et al., 2012).

### Preparation of KOK and Its Compositions

KOK (Gaepung Kyungokko®; Lot No. SQ12, 1,200 g) was obtained from Kwang Dong Pharmaceutical Company (Pyongtaek, Republic of Korea). It was manufactured as described previously (Cai et al., 2011; Jang et al., 2014; Lee et al., 2016b). Briefly, juice from the root of Rehmannia glutinosa Liboschitz var. purpurae Makino (32.0 g), powder of dried fruit of Lycium chinense Miller (0.9 g), powder of resin of Aquillaria agallocha Roxburgh (0.1 g), powder of cortex of Poria cocos Wolf (8.0 g), powder of dried root of Panax ginseng Meyer (2.8 g), honey (38.5 g of native acacia honey), and simple syrup (17.7 g of 85% sucrose solution) were mixed and heated in a water bath at 80°C for 72 h. The resulting viscous extract was stored at 4°C in a sealed jar. The KOK was standardized with 5-hydroxymethylfurfural (9.4%) for consistency of quality by the Kwang Dong Pharmaceutical Company (Cai et al., 2011; Jang et al., 2014; Lee et al., 2016b). The original formula of KOK consists of juice from the root of Rehmannia glutinosa Liboschitz var. purpurae Makino (9.6 g), powder of dried fruit of Poria cocos Wolf (1.8 g), powder of dried root of Panax ginseng C.A. Meyer (0.9 g), and honey (6 g) (Huh, 1610). However, KOK (Gaepung Kyungokko®) used in this study has a revised formula by Kwang Dong Pharmaceutical Company since 1974 (Korea Pharmaceutical Information Center, 2000; Korea Food and Drug Administration, 2007).

### Experimental Groups and Treatment With MPTP, KOK, and ML385

In order to determine the most effective dose of pre-treatment of KOK, mice were randomly divided into sham, MPTP, MPTP + KOK, and KOK groups. And to elucidate mechanism of KOK, mice were randomly divided into sham, MPTP, MPTP + KOK, MPTP + KOK + ML385, MPTP + ML385, KOK, and ML385 groups. MPTP intoxication was performed as previous described (Jackson-Lewis and Przedborski, 2007; Choi et al., 2018a). Briefly, mice in the MPTP and MPTP + KOK groups received four i.p. intoxications of MPTP-hydrochloride (20 mg/kg body weight; Sigma-Aldrich, St. Louis, MO, USA) dissolved in phosphate buffered saline (PBS) for 2 h intervals. KOK was dissolved in physiological saline and was treated orally at doses of 1 and 2 g/kg once daily for 12 days from 5 days before the first MPTP intoxication. ML385 (Nrf2 inhibitor; Medchem Express, Monmouth Junction, NJ, USA) was dissolved in PBS with 5% DMSO and was i.p. treated at dose of 30 mg/kg 30 min before KOK or saline treatment for 12 days. According to the Method of Ingestion obtained from Kwang Dong Pharmaceutical Company, the acceptable dose is a tea spoon (about 5 g) per day for adults. The acceptable dose to mice was determined based on well-known formula (Reagan-Shaw et al., 2008) with data from a preliminary experiment for this study and the best effective dose obtained from our previous studies (Jang et al., 2014; Lee et al., 2016b). Mice in the sham and KOK groups received saline instead of MPTP or KOK.

### Behavioral Assessment

To examine motor coordination, mice (*n* = 7 per group) were subjected to pole and rotarod tests as previous described (Choi et al., 2018a). The nest building behavior was measured as an indicator of health and welfare in mice as previous described (Choi et al., 2018a). The behavioral tests were performed by an experimenter who was unaware of the experimental conditions and was done under constant temperature (23 ± 2°C) and humidity (55 ± 5%) in a quiet room, 1 day before and 1,3,5, and 7 days after MPTP intoxication.

### Western Blot Assays

At 7 days following the last MPTP intoxication, coronal brain slices (3 mm in thickness) from each mouse (*n* = 3 per group) were prepared as previous described (Choi et al., 2018a) and SNpc and striatum regions were sampled using microscissors and blade under a dissection microscope. Western blot assay was performed as previously described (Choi et al., 2018a,c). Briefly, the polyvinylidene fluoride (PVDF) membrane strips with a specific protein from SNpc and striatum were probed overnight with rabbit anti-tyrosine hydroxylase (1:1,000; Millipore, Bedford, MA, USA), rabbit anti-ionized calcium binding adapter molecule-1 (Iba-1; 1:500; WAKO, Osaka, Japan), mouse anti-glial fibrillary acidic protein (GFAP; 1:1,000; Millipore), rat anti-platelet endothelial cell adhesion molecule-1 [PECAM-1 (CD31); 1:500; Santa Cruz Biotechnology, Santa Cruz, CA, USA], rabbit anti-phospho (p)-extracellular signal-regulated kinase (ERK), rabbit anti-p-c-Jun N-terminal kinase (JNK), rabbit anti-p-p38, rabbit anti-nuclear factor kappa-light-chain-enhancer of activated B cells (NF-κB) p65, rabbit anti-p-IκBα (1:1,000; Cell Signaling Technology, Beverly, MA, USA), mouse anti-Kelch-like ECH-associated protein 1 (Keap1; 1:1,000, Santa Cruz Biotechnology), rabbit anti-nuclear factor erythroid 2-related factor 2 (Nrf2; 1:1,000, Santa Cruz Biotechnology), mouse anti-heme oxygenase-1 (HO-1; 1:1,000; Enzo Life Sciences, Farmingdale, NY, USA), or mouse anti-NAD(P)H dehydrogenase [quinone] 1 (NQO1; 1:1,000; Cell Signaling Technology) at 4°C. To investigate nuclear translocation of Nrf2 and cytosolic expression of NQO-1, both nucleus and cytosol proteins isolated from SNpc and striatum were used. PVDF membranes were then incubated with horseradish peroxidase-conjugated secondary antibody to enhance chemiluminescence analysis (Amersham Pharmacia Biotech, Piscataway, NJ, USA) and visualized using a super cooled-CCD camera system with a Davinch-K Gel imaging system (Dvinch-K, Seoul, Republic of Korea). For normalization of antibody signals, the PVDF membranes were stripped and reprobed with rabbit anti-histone H3 (1:5,000; Cell Signaling Technology), rabbit anti-glyceraldehyde 3-phosphate dehydrogenase (GAPDH; 1:5,000; Cell Signaling Technology), or total antibody against each protein (1:2,000; rabbit anti-ERK, rabbit anti-JNK, rabbit anti-p38, rabbit anti-NF-κB p65, and mouse anti-IκBα; Cell Signaling Technology). Western blot assay was performed at least three times. The density of each band was converted to a numerical value using the Photoshop CS2 program (Adobe, San Jose, CA, USA) after subtracting background values from an area of film immediately adjacent to the stained band. Data are expressed as the ratio of each expression against GAPDH, total protein, or histone H3 in each sample.

### Immunohistochemical Assay

At 7 days following the last MPTP intoxication, brain samples from each group (*n* = 5 per group) were acquired by perfusion using ice cold 4% paraformaldehyde solution as previously described (Choi et al., 2018c). Sequential coronal sections (30 μm thickness) of these brain samples were acquired using a model CM3050S freezing microtome (Leica Biosystems, Wetzlar, Germany) from the level of the SNpc (bregma −2.54 to −3.40 mm) and mid-striatum (bregma +0.26 to +1.10 mm) according to the mouse brain atlas (Franklin and Paxinos, 2008). Immunohistochemical assay was accomplished as previously described (Lee et al., 2016a; Choi et al., 2018a,c). Briefly, the sections (*n* = 3 per brain) from all groups were incubated with either rabbit anti-TH (1:1,000; Millipore) or rabbit anti-Iba-1 (1:2,000; WAKO), followed by incubation with biotinylated rabbit IgG antibody (1:200; Vector Laboratories, Burlingame, CA, USA) and avidin–biotinylated horseradish peroxidase complex (1:200; Vector Laboratories). The sections were visualized with 3,3′-diamino-benzidine and cover-slipped with Permount.

For double immunofluorescent staining, sections were incubated overnight at 4°C with a mixture of mouse anti-PECAM-1 (1:500; Santa Cruz Biotechnology) and rabbit anti-GFAP (1:1,000; Millipore) antibodies or a mixture of rabbit anti-Nrf2 (1:500; Santa Cruz Biotechnology) and mouse anti-NQO1 (1:1,000; Cell Signaling Technology) antibodies. The sections were then incubated for 1 h at room temperature with a mixture of Cy3- and FITC conjugated mouse/rabbit IgG antibody (1:200; Jackson ImmunoResearch, West Grove, PA, USA) and then examined with confocal imaging system (LSM 5 PASCAL; Carl Zeiss, Germany).

### Analysis of MitoSOX Activity

MitoSOX activity was measured as previously described (Wojtala et al., 2014). Briefly, free-floating sections from each group (sham, *n* = 3; MPTP, *n* = 5; MPTP + KOK 2, *n* = 5, and KOK 2, *n* = 3) were incubated for 10 min, to allow the probe to enter the cell and start the reaction within the mitochondria, at 37°C in 0.5 ml of measurement buffer containing 5 mM MitoSOX Red (Molecular Probes, Eugene, OR, USA). Subsequently, the sections were washed twice with PBS and coverslipped.

### Real-Time Polymerase Chain Reaction (PCR) Analysis

At 7 days following MPTP intoxication, SNpc, and striatal regions (*n* = 5 per group) were removed for immunoblot blot assays. Real-time PCR assay was performed as described previously (Lee et al., 2016a). The mRNA levels of each target gene were normalized to that of GAPDH mRNA. Fold-induction was calculated using the 2–ΔΔCT method as previously described (Livak and Schmittgen, 2001). The primer sequences were as follows; interleukin (IL)-6-5′-TCC ATC CAG TTG CCT TCT TGG-3′ and 5′-CCA CGA TTT CCC AGA GAA CAT G-3′, tumor necrosis factor (TNF)-α-5′-AGC AAA CCA CCA AGT GGA GGA-3′ and 5′-GCT GGC ACC ACT AGT TGG TTG T-3′, Cyclooxygenase (COX)-2-5′-CAG TAT CAG AAC CGC ATT GCC-3′ and 5′-GAG CAA GTC CGT GTT CAA GGA-3′, inducible nitric oxide synthase (iNOS)-5′-GGC AAA CCC AAG GTC TAG GTT-3′ and 5′-TCG CTC AAG TTC AGC TTG GT-3′, endothelial intercellular adhesion molecule (ICAM)-1-5′-TGC GTT TTG GAG CTA GCG GAC CA-3′ and 5′-CGA GGA CCA TAC AGC ACG TGC AG-3′, vascular cell adhesion molecule (VCAM)-1-5′-CCT CAC TTG CAG CAC TAC GGG CT-3′ and 5′-TTT TCC AAT ATC CTC AAT GAC GGG-3′, occludin-5′-ATG CAT CTC TCC GCC ATA CAT-3′ and 5′-AGA CCT GAT GAA TTC AAA CCC AAT-3, zoula occludens (ZO)-1-5′-AAG GCA ATT CCG TAT CGT TG-3′ and 5′-CCA CAG CTG AAG GAC TCA CA-3′, and GAPDH-5′-AGG TCA TCC CAG AGC TGA ACG-3′ and 5′-CAC CCT GTT GCT GTA GCC GTA T-3′.

### Statistical Analyses

All data are presented as means ± S.E.M. Statistical analyses were performed using the SPSS 23.0 package (SPSS Inc, Chicago, IL, USA) for Windows. Two-sample comparisons were carried out using the Student's *t*-test and multiple comparisons were made using one-way ANOVA with Tukey's *post-hoc* test. Statistical difference was identified at the 5% level unless otherwise indicated.

## Results

### Mitigating Effects of KOK on Neurological Disorders Following MPTP Intoxication

**Figure 1:**
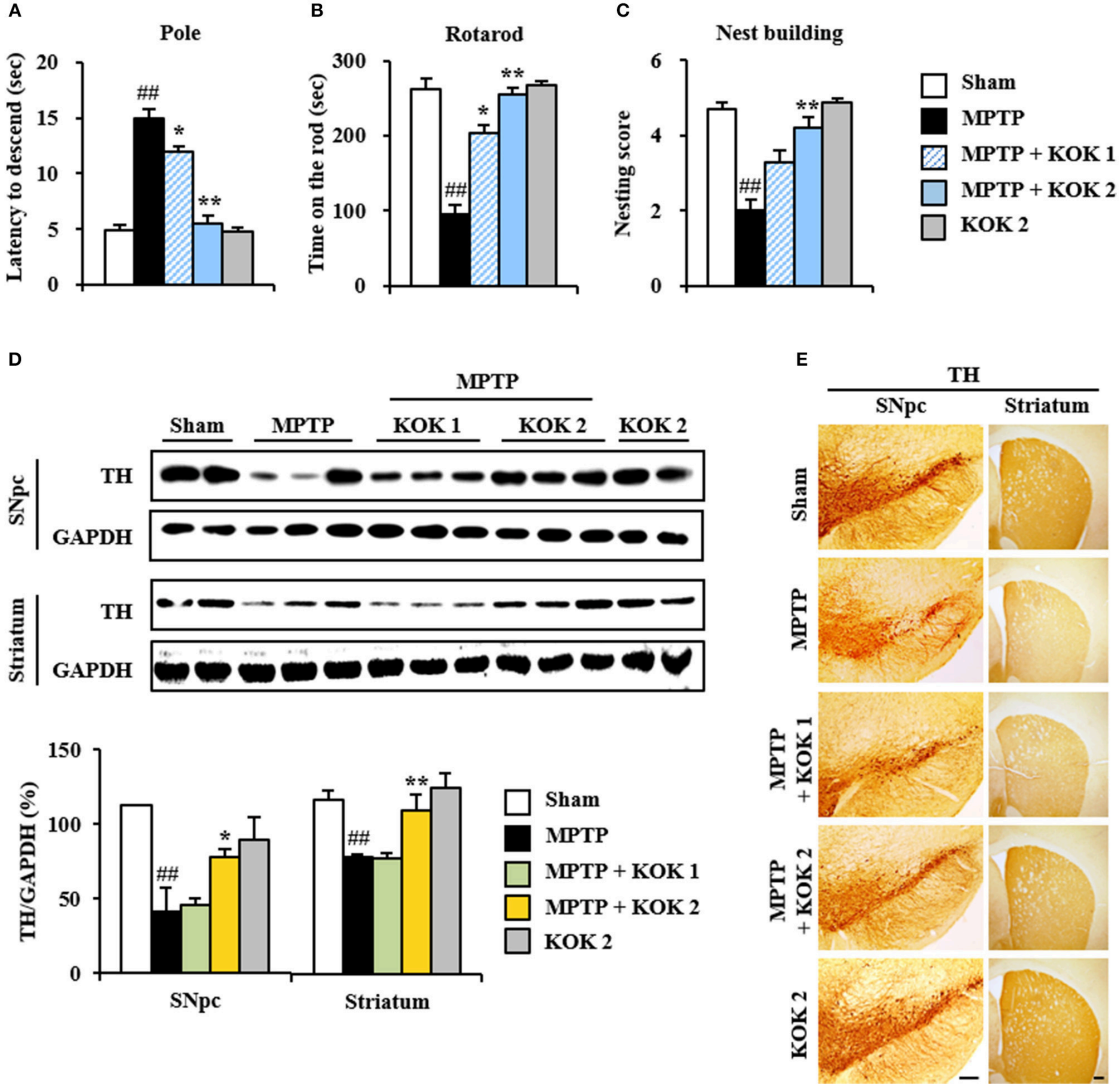
KOK mitigates neurological dysfunctions and dopaminergic neuronal loss by MPTP intoxication**. (A–C)** Mice (*n* = 7 per group) were orally received KOK (1 and 2 g/kg/day) or saline from for 12 days from 5 days before the first MPTP intoxication. Pole test **(A)**, rotarod performance test **(B)**, and nest building behavior test **(C)** were accomplished 3, 3, and 1 days after the last MPTP intoxication. **(D)** SNpc and striatum from all groups (*n* = 2–3 per group) were acquired 7 days after the last MPTP intoxication and quantitatively measured by Western blot assay using TH antibody. The upper panels illustrate representative Western blots. **(E)** SNpc and striatum sections (*n* = 3 per brain) from each group (*n* = 5 per group) were acquired 7 days after the last MPTP intoxication and immunostained with TH antibody. Scale bar = 100 μm. ANOVA test; ##*p* < 0.01 vs. Sham group; ^*^p < 0.05 and ^**^p < 0.01 vs. MPTP group.

First, we investigated mitigating effects of KOK on MPTP-induced neurological dysfunction using pole and rota rod tests to assess motor function. Total time taken in the pole test was increased by 206.1% (15.0 ± 0.8 s) in the MPTP group compared to that in the sham group (4.9 ± 0.4 s) while the total time was significantly reduced (by 20.0–63.3%) after KOK treatment (12.0 ± 3.4 and 5.5 ± 0.3 s for KOK at 1 and 2 g/kg/day, respectively; Figure 1A). In rotarod performance test, the average latency to fall was reduced by 63.9% (94.4 ± 19.2 s) in the MPTP group compared to that in the sham group (261.8 ± 94.4 s) while the average latency was significantly increased by 115.3–169.4% after KOK treatment (203.2 ± 15.7 and 254.3 ± 11.6 s for KOK at 1 and 2 g/kg/day, respectively; Figure 1B). We also performed nest building behavior test as an indicator of health and welfare. The mean score of the quality of built nest was reduced by 57.5% (2.0 ± 0.3) in the MPTP group compared to that in the sham group (4.7 ± 0.2) while the mean score was significantly increased by 65%-110% following KOK treatment (3.3 ± 0.3 and 4.2 ± 0.3 in for KOK at 1 and 2 g/kg/day, respectively; Figure 1C) compared to that of the MPTP group (Figure 1C). KOK did not significantly alter neurological behavior. At the end of the experiment, survival rates of mice in sham, MPTP, MPTP + KOK 1 g/kg, MPTP + KOK 2 g/kg, and KOK 2 g/kg groups were 100, 83.3, 91.6, 100, and 100%, respectively (data not shown). These results indicate that KOK might be able to reduce MPTP-induced neurological impairments.

### Mitigating Effects of KOK on Dopaminergic Neuronal Loss in the SNpc Following MPTP Intoxication

Motor manifestations in PD and MPTP-induced animal model are primarily linked to SNpc and depletion of the dopamine in the striatum (Brichta et al., 2013; Gubellini and Kachidian, 2015). Thus, we examined whether KOK might have protective effect against the loss of dopaminergic neurons/fibers at 7 days after MPTP intoxication. Results of immunoblotting assay (Figures 1D–E) revealed that expression levels of TH protein in SNpc and striatum were decreased in the MPTP group (41.5 ± 15.7 and 79.4 ± 1.2%, respectively) compared to those in the sham group (112.3 ± 0.6 and 117.4 ± 6.5%, respectively). However, KOK treatment (2 g/kg/day) significantly inhibited such decrease (77.6 ± 5.6 and 110.8 ± 11.1% for KOK treatment at 1 and 2 g/kg/day, respectively) (Figure 1D). These results were consistent with changes in intensity of TH immunoreactivity (Figure 1E). Majority of fibers of dopaminergic neurons in the SNpc are known to project to the striatum while dopaminergic neuronal loss can lead to the reduction of dopamine in the striatum (Shulman et al., 2011; Kalia and Lang, 2016; Poewe et al., 2017). Thus, we measured changes of TH expression in the striatum. In agreement with results in the SNpc, TH protein expression levels were decreased by 45.5 ± 3.4% in the striatum of MPTP group compared to those in the sham group (45.5 ± 3.4%). However, KOK treatment at 2 g/kg/day) significantly inhibited such decrease (52.7 ± 3.9 and 72.8 ± 12.3% for KOK at 1 and 2 g/kg/day, respectively) (Figure 1D), consistent with changes of intensity of TH immunoreactivity in the striatum (Figure 1E). However, KOK treatment alone did not produce significant changes in TH expression in the SNpc or striatum. These results suggest that KOK might have positive effects on MPTP-mediated neurological dysfunction by reducing dopaminergic neuronal loss.

### Anti-inflammatory Effect of KOK on SNpc and Striatum Following MPTP Intoxication

**Figure 2:**
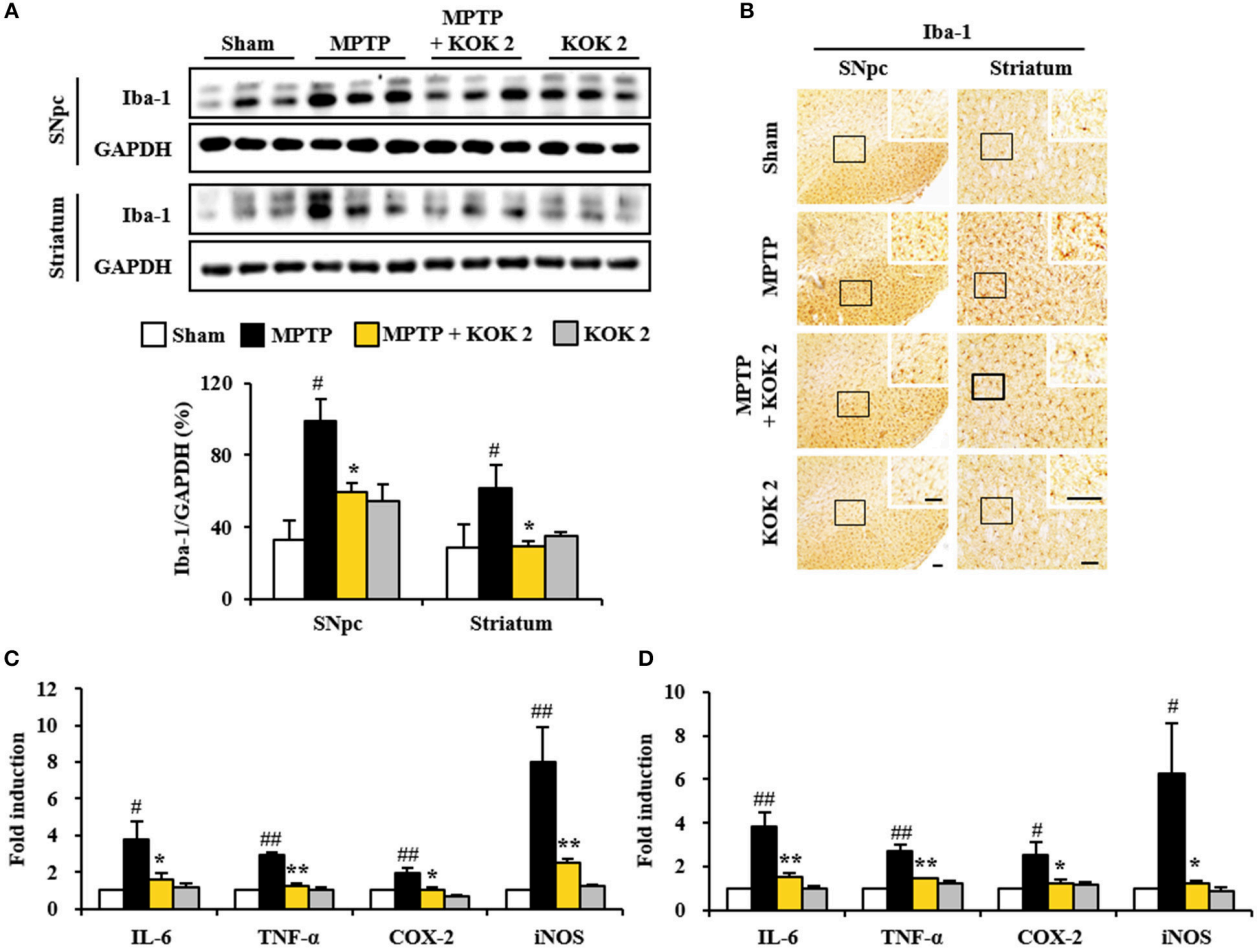
KOK inhibits microglial activation and inflammatory mediators in the SNpc and striatum by MPTP intoxication**. (A)** Seven days after the last MPTP intoxication, SNpc and striatum from all groups (*n* = 3 per group) were measured by Western assay using Iba-1 antibody. The left panels illustrate representative Western blots. **(B)** Seven days after the last MPTP intoxication, SNpc, and striatum sections (*n* = 3 per brain) were immunostained with Iba-1 antibody. Insets display high magnification micrographs of the areas marked with squares. **(C,D)** Seven days after MPTP intoxication, real-time PCR assay was performed to measure mRNA expression of IL-6, TNF-α, COX-2, and iNOS in SNpc **(C)** and striatum **(D)** (*n* = 3 per group). Scale bar = 50 μm. ANOVA test; #*p* < 0.05 and ##*p* < 0.01 vs. Sham group; ^*^p < 0.05 and ^**^p < 0.01 vs. MPTP group.

Inflammatory response is known to be associated with neurological disorders (Liberatore et al., 1999; Lobsiger and Cleveland, 2007; Du et al., 2017). Thus, we evaluated whether KOK might exert anti-inflammatory effect and whether such effect was associated with anti-neuronal death. The expression of Iba-1 protein (a marker for microglia) was increased in SNpc (98.7 ± 12.7%) and striatum (61.7 ± 12.2%) of the MPTP group compared to that in the sham group (33.1 ± 10.9% in the SNpc and 28.6 ± 13.2% in the striatum). However, KOK treatment (2 g/kg) significantly inhibited such increase (59.5 ± 5.4% in the SNpc and 29.8 ± 2.5% in the striatum) compared to that in the MPTP group (Figure 2A). These changes of Iba-1 protein levels were consistent with the intensity of Iba-1-immunoreactivity (Figure 2B). Iba-1-immunoreactive cells showed typically activated form in the SNpc and striatum in the MPTP group. However, Iba-1-immunoreactive cells from KOK-treated group showed resting form (Figure 2B).

Activated microglia in the SNpc and striatum may produce inflammatory mediators known to be involved in degeneration of dopaminergic neurons (Liberatore et al., 1999; Lobsiger and Cleveland, 2007; Du et al., 2017). Thus, we determined regulating effect of KOK on the expression of inflammatory mediators. Based on real-time PCR assay, relative mRNA expression levels of representative cytokines IL-6 and TNF-α, COX-2, and iNOS were upregulated 3.8-, 2.9-, 1.9-, and 8.0-fold, respectively, in the SNpc (Figure 2C). They were upregulated 3.8-, 2.7-, 2.5-, and 6.3-fold, respectively, in the striatum (Figure 2D), at 7 days after MPTP intoxication compared to those in the sham group. However, KOK treatment (2 g/kg) significantly prevented their upregulation in the SNpc (57.9, 85.0, 42.1, and 68.8%, respectively, Figure 2C) and striatum (60.5, 48.2, 52.0, and 81.0%, respectively, Figure 2D) induced by MPTP intoxication. These findings suggest that KOK might mitigate MPTP-induced neurotoxicity by inhibiting microglial activation and inflammatory response.

### Protective Effect of KOK on BBB Integrity Following MPTP Intoxication

**Figure 3:**
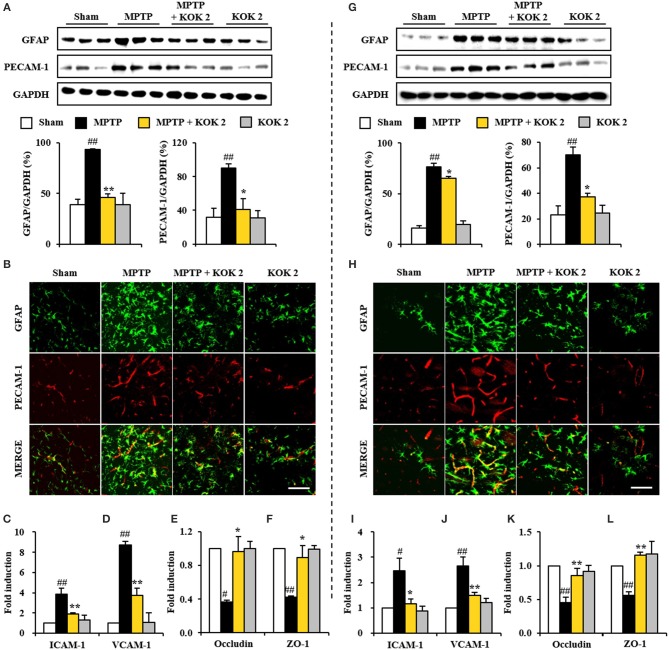
KOK exerts protective effect against the BBB disruption in the SNpc **(A–F)** and striatum **(G–L)** following MPTP intoxication. **(A,G)** Seven days after the last MPTP intoxication, SNpc and Striatum (*n* = 3 per group) were performed by Western blot assay using PECAM-1 and GFAP antibodies. The upper panels illustrate representative Western blots. **(B,H)** Seven days after the last MPTP intoxication, SNpc and Striatum (*n* = 3 per brain) from all groups (*n* = 5 per group) were performed by immunofluorescence using PECAM-1 and GFAP antisera. Scale bar = 50 μm. **(C–F,I–L)** Real-time PCR using primers for ICAM-1 **(C,I)**, VCAM-1 **(D,J)**, occludin **(E,K)**, and ZO-1 **(F,L)** was used to quantify the expression of these molecules in SNpc **(C–F)** and striatum **(I–L)** (*n* = 3 per group) 7 days after MPTP-intoxication. ANOVA test; #*p* < 0.05 and ##*p* < 0.01 vs. Sham group. ^*^p < 0.05 and ^**^p < 0.01 vs. MPTP group.

Disruption of blood-brain barrier (BBB) plays an important role in cellular damage of neurological diseases including PD (Abbott et al., 2006; Sandoval and Witt, 2008; Zlokovic, 2008). Thus, we explored whether KOK could exert positive effect on the maintenance of BBB integrity. At 7 days after MPTP intoxication, protein expression levels of PECAM-1, a marker of BBB disruption, were increased in the SNpc (90.0 ± 5.3%) and striatum (70.0 ± 3.0%) of the MPTP group compared to those in the sham group (32.0 ± 10.1% in the SNpc and 23.3 ± 6.9% in the striatum). However, KOK treatment at 2 g/kg prevented such increase in its expression (40.9 ± 13.0% in the SNpc and 37.1 ± 3.0% in the striatum) (Figures 3A,G), in accordance with alteration of PECAM-1 immunoreactivity (Figures 3B,H). Expression levels of GFAP protein, one of main components of BBB (Abbott et al., 2006), were slightly increased in the SNpc (93.3 ± 0.5%) and striatum (76.5 ± 3.5%) of the MPTP group compared to those in the sham group (38.8 ± 5.8% in the SNpc and 16.1 ± 2.3% in striatum). However, KOK treatment at 2 g/kg inhibited such increase in its expression (46.3 ± 3.6% in the SNpc and 65.3 ± 1.6% in striatum) (Figures 3A,G), in accordance with alteration of GFAP-immunoreactivity. Interestingly, PECAM-1 immunoreactivity was observed in GFAP-immunoreactive structures (Figures 3B,H). Successively, we explored whether KOK might inhibit changes of adhesion and junctional molecules. Based on real-time PCR assay, mRNA expression levels of ICAM-1 and VCAM-1 as representative adhesion molecules were increased in the SNpc (3.8- and 8.7-fold, respectively) and striatum (2.5- and 2.6-fold, respectively) after MPTP intoxication compared to those in the sham group while KOK treatment blocked such increases (50.0 and 57.5%, respectively, in the SNpc and 52.0 and 42.3%, respectively, in the striatum) (Figures 3C,D,I,J). However, mRNA expression levels of occludin and ZO-1 as representative junctional molecules were decreased in the SNpc (0.4- and 0.4-fold, respectively) and striatum (0.5- and 0.6-fold, respectively) after MPTP intoxication while KOK treatment inhibited such decrease (150.0 and 125.0% for occludin and ZO-1, respectively, in the SNpc and 60.0 and 100.0%, respectively, in the striatum) (Figures 3E,F,K,L). These findings suggest that the protective effect of KOK on BBB integrity might contribute to its protective effect on MPTP-induced neurotoxicity.

### Inhibiting Effect of KOK on MAPKs and NF-κB Pathways in SNpc and Striatum After MPTP Intoxication

**Figure 4:**
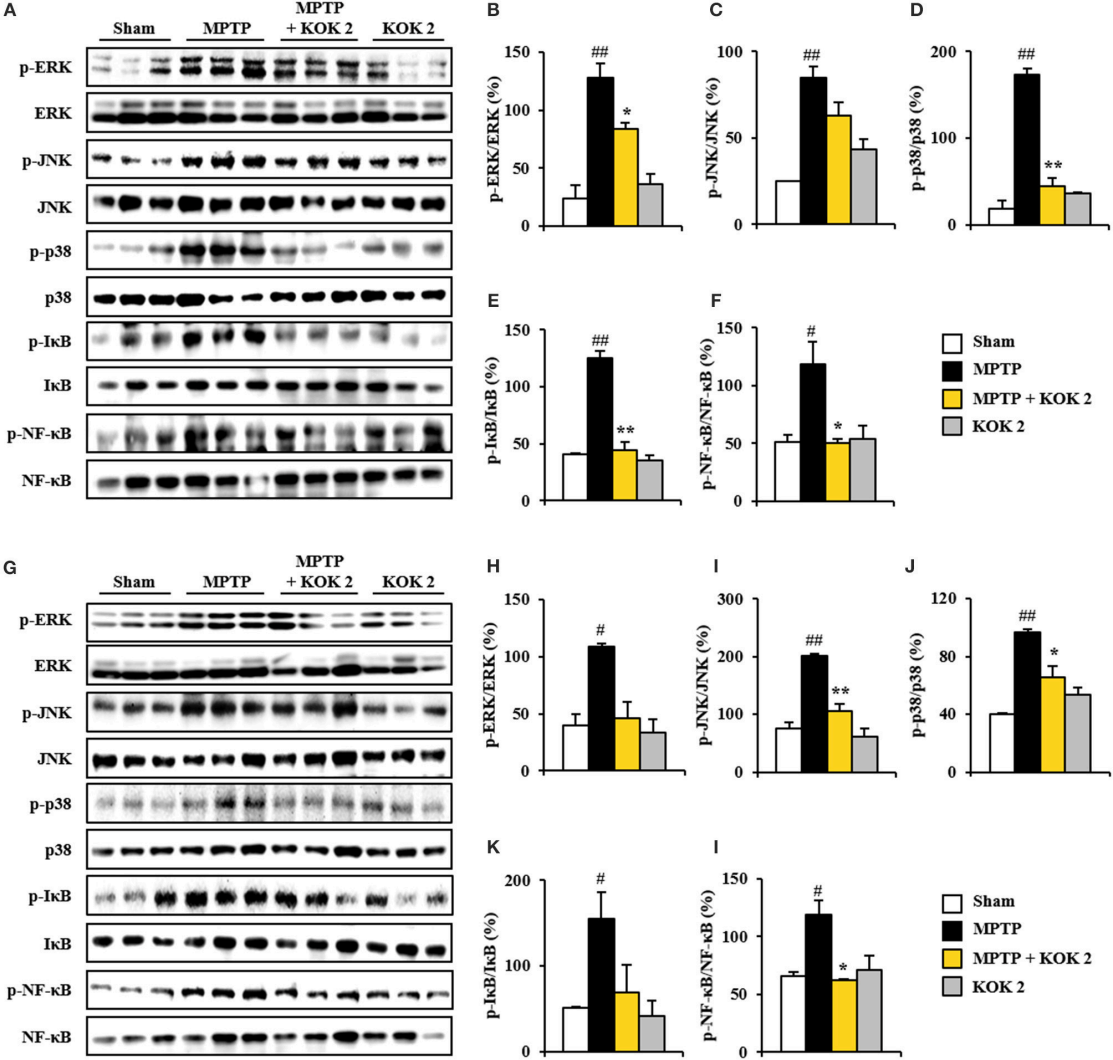
KOK blocks MAPKs and NF-κB signaling pathways in the SNpc and striatum following MPTP intoxication. **(A–L)** Seven days after MPTP intoxication, SNpc, and striatum sample from all groups (*n* = 3 per group) were performed by Western blot assay to quantify the changes in the MAPKs **(A–D, G–J)** and NF-κB pathways **(A, E–G,K,L)**. SNpc **(A–F)** and striatum **(G–L)**. The left panels illustrate representative Western blots **(A,G)**. ANOVA test; #*p* < 0.05 and ##*p* < 0.01 vs. Sham group. ^*^p < 0.05 and ^**^p < 0.01 vs. MPTP group.

MAPKs (ERK, JNK, and p38) and NF-κB pathways are pivotal signaling pathways in neuronal loss, BBB disruption, inflammatory response, and oxidative stress (Abbott et al., 2006; Sandoval and Witt, 2008; Zlokovic, 2008). Thus, we tested whether KOK could positively regulate these signaling pathways in the SNpc and striatum following MPTP intoxication. Based on immunoblot assay, expression levels of p-ERK, p-JNK, and p-p38 proteins were significantly increased in the SNpc (127.8 ± 6.8, 84.9 ± 6.2, and 173.3 ± 7.0%, respectively) and striatum (108.6 ± 3.2, 201.3 ± 3.7, and 96.6 ± 2.6%, respectively) at 7 days after MPTP intoxication compared to those in the sham group (24.0 ± 9.3, 24.9 ± 0.3, and 18.5 ± 9.5%, respectively, in the SNpc, and 39.3 ± 10.1, 75.6 ± 10.9, and 40.1 ± 0.7%, respectively). However, KOK treatment at 2 g/kg significantly inhibited these increases (83.5 ± 0.6, 63.0 ± 7.7, and 44.2 ± 9.3%, respectively, in the SNpc, and 46.0 ± 14.0, 106.4 ± 11.3, and 65.2 ± 8.4%, respectively, in striatum) (Figures 4A–D,G–J). Successively, we investigated the effect of KOK on NF-κB pathway in the SNpc and striatum after MPTP intoxication. Expression levels of p-IκB and p-NF-κB were significantly increased in the SNpc (125.2 ± 5.7 and 118.6 ± 18.9%, respectively) and striatum (154.5 ± 31.5 and 118.9 ± 12.0%, respectively) at 7 days following MPTP intoxication compared to those in the sham group (40.4 ± 1.3 and 50.9 ± 6.6%, respectively, in the SNpc, and 50.7 ± 1.3 and 65.6 ± 3.7%, respectively, in the striatum). However, treatment with 2 g/kg KOK significantly inhibited such increases (44.7 ± 7.2 and 50.0 ± 3.6%, respectively, in the SNpc, and 69.2 ± 31.3 and 62.7 ± 0.2%, respectively, in the striatum) (Figures 4A, E–G,K,L). KOK treatment alone did not significantly affect MAPKs or NF-κB pathways (Figure 4). These findings indicate that KOK might mitigate MPTP-mediated neurotoxicity via blocking representative signaling pathways (MAPKs and NF-κB pathways) involved in inflammation.

### Activating Effect of KOK on Keap1-Nrf2 Pathway in SNpc and Striatum After MPTP Intoxication

**Figure 5:**
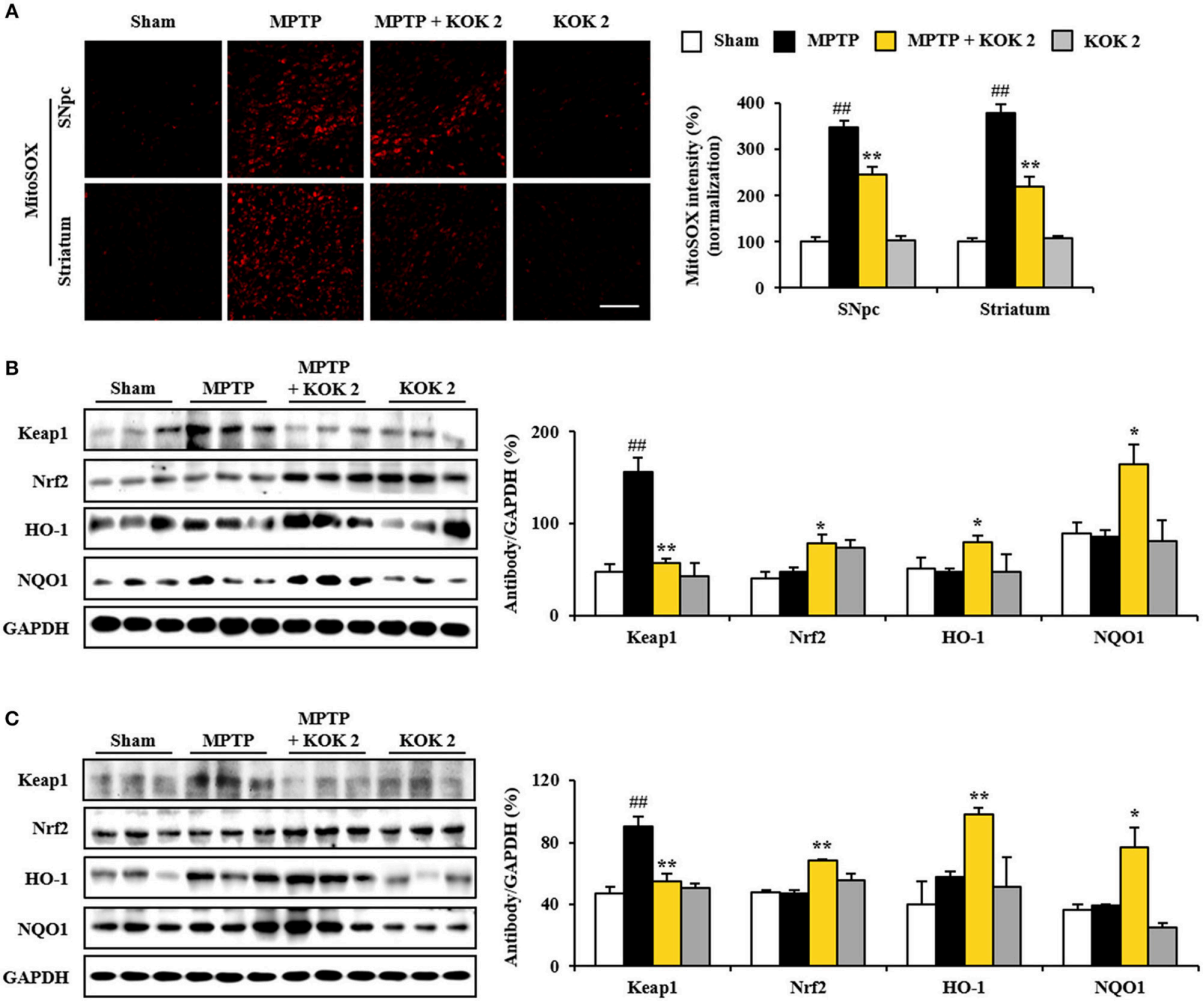
KOK up-regulates Keap1-Nrf2 signaling in the SNpc and striatum following MPTP intoxication. **(A)** At 7 days after the last MPTP intoxication, SNpc and striatal sections (*n* = 5 per group) were subjected to MitoSOX staining. The staining intensity was then quantified. Insets display high magnification micrographs of areas marked with squares. **(B,C)** Seven days after the last MPTP intoxication, SNpc, and striatum sample (*n* = 3 per group) were performed by Western blot assay. Keap1, Nrf2, HO-1, and NQO1. SNpc **(B)** and striatum **(C)**. The top panels illustrate representative Western blots. ANOVA test; ##*p* < 0.01 vs. Sham group. ^*^p < 0.05 and ^**^p < 0.01 vs. MPTP group.

MPTP-induced mitochondrial perturbation is known to be associated with high production of reactive oxygen species (ROS). Thus, we measured ROS generation using MitoSOX staining. The intensity of MitoSOX was increased in the SNpc (347.5 ± 14.6%) and the striatum (378.2 ± 18.7%) of the MPTP group compared to that of the sham group (100.0 ± 10.9% in the SNpc and 100.0 ± 7.5% in the striatum). However, such increase in intensity was inhibited (245.3 ± 16.1 and 218.5 ± 23.0%) by treatment with KOK at 2 g/kg (Figure 5A). Activation of Keap1-Nrf2 antioxidant pathway has beneficial effects for neurodegenerative disorders such as PD in animal models (Blesa et al., 2015; Todorovic et al., 2016; Lee Y. M. et al., 2017). Thus, we explored whether positive effects of KOK (Figures 1–3) might be linked to up-regulation of Keap1-Nrf2 pathway. Based on immunoblot blot assay, levels of Keap1 (a repressor protein that binds to Nrf2) activation in SNpc (156.2 ± 15.5%) and striatum (90.1 ± 6.3%) following MPTP intoxication were significantly increased compared to those in the sham group (48.0 ± 7.6% in the SNpc and 46.7 ± 4.3% in the striatum). KOK treatment (2 g/kg) further decreased expression levels of Keap1 (57.2 ± 4.9% in the SNpc and 54.9 ± 4.7% in the striatum) (Figures 5B,C). As expected, expression levels of Nrf2 transcription factor in the SNpc (48.0 ± 3.8%) and striatum (47.0 ± 2.4%) following MPTP intoxication were similar to those in the sham group (40.5 ± 6.9% in the SNpc and 47.7 ± 1.8% in the striatum). KOK treatment (2 g/kg) further increased its expression (78.7 ± 9.3% in the SNpc and 68.2 ± 0.7% in the striatum) (Figures 5B,C). Successively, we determined the effect of KOK on expression levels of Nrf2 targeting genes. Expression levels of HO-1 and NQO-1 were increased by 80.1 ± 7.5 and 165.2 ± 21.0%, respectively, in the SNpc. They were increased 98.1 ± 4.0 and 76.5 ± 13.3%, respectively, in the striatum of KOK-treated group compared to those in the MPTP group (47.6 ± 3.3 and 86.0 ± 7.1%, respectively, in the SNpc, and 57.6 ± 3.8 and 39.2 ± 0.8%, respectively, in striatum) (Figures 5B,C). These results indicate that antioxidant activity of KOK might have contributed to its mitigating activities on MPTP-mediated neurotoxicity.

### Neutralizing Effect of Nrf2 Inhibitor Against Protective Effect of KOK in Neurological Dysfunctions and Activated MAPKs and NF-κB Signaling Following MPTP Intoxication

**Figure 6:**
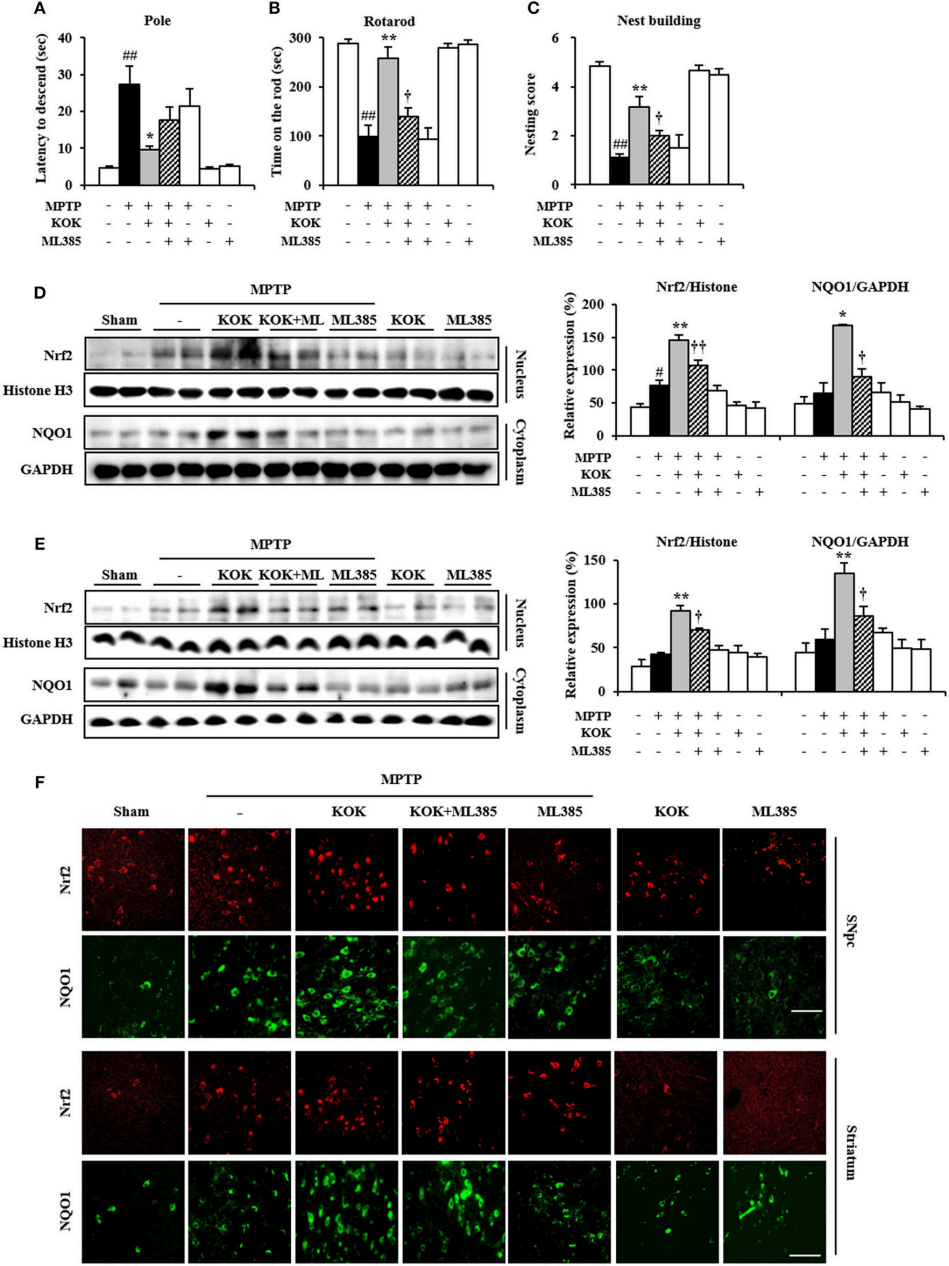
Nrf2 inhibitor neutralizes the protective effect of KOK following MPTP intoxication**. (A–C)** Nrf2 inhibitor (ML385; 30 mg/kg, i.p.) or saline was i.p. injected to mouse once daily from 30 min before KOK treatment in MPTP-intoxication model (*n* = 4–6 per group). Pole test **(A)**, rotarod performance test **(B)**, and nest building behavior test **(C)** were accomplished at 5, 7, and 1 days, respectively, after the last MPTP intoxication. **(D,E)** Seven days after the last MPTP intoxication, nucleus, and cytosol isolated from SNpc **(D)** and striatum **(E)** from all groups (*n* = 4 per group) were subjected to Western blot assay to quantify changes in Nrf2 nuclear translocation and NQO1 expression, respectively. **(F)** Seven days after the last MPTP intoxication, SNpc and striatal sections (*n* = 3 per brain) were subjected to immunofluorescence staining using Nrf2 and NQO1 antibodies. Scale bar = 50 μm. ANOVA test; #*p* < 0.05 and ##*p* < 0.01 vs. Sham group; ^*^p < 0.05 and ^**^p < 0.01 vs. MPTP group; †*p* < 0.05, ††*p* < 0.01 vs. MPTP+KOK group.

In the present study, pretreatment with KOK significantly activated Nrf2 signaling associated with inhibition of MAPKs and NF-κB signaling, mitigation of neurological disorders, and reduction of dopaminergic neurodegeneration and inflammation in the SNpc and striatum after MPTP intoxication (Figures 1–5). These results suggest that pre-inhibiting Nrf2 pathway might neutralize the protective effect of KOK against MPTP-induced neurotoxicity. To test this possibility, we i.p. injected mice with Nrf2 inhibitor (ML385) 30 min before KOK or saline treatment in MPTP-intoxicated animal model (Figures 6A–C). As expected, the positive effect of KOK for neurological impairment by MPTP-intoxication (9.6 ± 0.9 s in pole test, 258.3 ± 22.4 s in rotarod test, and 3.2 ± 0.4 in nest building test) was significantly neutralized by pre-inhibiting Nrf2 signaling using 30 mg/kg of Nrf2 inhibitor (17.6 ± 3.6 s in pole test, 139.8 ± 16.9 s in rotarod test, and 2.0 ± 0.5 s in nest building test; Figures 6A–C). Enhanced expression of Nrf2 in nucleus and NQO1 in cytoplasm by KOK (88.3 and 158.0% in SNpc and 119.3 and 128.2% in striatum) was also neutralized by pre-inhibiting using Nrf2 inhibitor (26.1 and 46.5% in SNpc and 23.8 and 35.8% in striatum; Figures 6D,E). In agreement with these results, increased immunoreactivity of Nrf2 in nucleus and NQO1 in cytoplasm by KOK was neutralized by pre-inhibiting using Nrf2 inhibitor (Figure 6F). Nrf2 inhibitor itself did not significantly affect the neurological score or activity of Nrf2 and NQO1 in mice (Figures 6A–F). These results provide evidence that the neuroprotective effect of KOK after MPTP-intoxication is associated with the activation of Keap1-Nrf2 signaling.

## Discussion

The pathogenesis of idiopathic PD is thought to be multifactorial. It is associated with mitochondrial dysfunction, apoptosis, oxidative stress, and inflammation (Riess and Krüger, 1999; Shulman et al., 2011; Blesa et al., 2015; Kalia and Lang, 2016; Poewe et al., 2017). Such complexity challenges dominant paradigm in drug discovery or single-target drug for a single mechanism. Although this paradigm has achieved considerable success for some diseases, it has failed to provide effective approaches for PD. Currently, the desire to develop more effective and safer therapeutics for PD is shifting drug design toward multi-target compounds acting in the central nervous system that are designed based on natural products (Bajda et al., 2011; Dias and Viegas, 2014; Zheng et al., 2014; Calabresi and Di Filippo, 2015). Here, we demonstrate that KOK has multi-target activities including anti-motor disorder, anti-dopaminergic neuronal loss, anti-inflammation, anti-BBB disruption, and anti-oxidant. Thus, KOK might have potential to prevent and treat PD.

Neurological dysfunctions in PD patients and *in vivo* animal models are primarily related to dopaminergic neuronal loss by apoptosis in the SNpc and depletion of dopamine in the striatum (Brichta et al., 2013; Gubellini and Kachidian, 2015). KOK significantly attenuates ischemia-induced cognitive impairments in Y-maze and novel object recognition tasks. This is associated with its protective activity against reduction of hippocampal cell death in CA1 (Cai et al., 2011). KOK also reduces sperm loss, apoptosis protein expressions in the testes, and morphological abnormality of seminiferous tubules and seminiferous epithelium after heat-induced damage (Hwang et al., 2015). KOK can also reduce endometrial apoptosis in hyperandrogenized rats (Lee et al., 2016b). Additionally, components (fruits of Lycium chinense Miller, Poria cocos Wolf, and ginseng) of KOK have protective effects against apoptosis in oxidative stress-induced hepatotoxicity (Zhang et al., 2010), cisplatin-induced LLC-PK1 cells (Lee D. et al., 2017), and MPTP-induced MPTP model of PD (Choi et al., 2018a). Based on these reports, effects of KOK such as its anti-behavioral dysfunction and anti-dopaminergic neuronal death induced by apoptosis after MPTP intoxication may be explained, although the underlying mechanism remains unclear.

Microglia play pivotal roles in the development and maintenance of the brain micro-environment. They can be activated within or around lesions of neurodegenerative disorders such as PD. Activated microglia will produce pro-inflammatory and/or anti-inflammatory mediators (Lobsiger and Cleveland, 2007; Du et al., 2017). Thus, intensive research efforts have been made on regulation of microglial activation to protect dopaminergic neurons in *in vivo* model of PD and PD patients (Du et al., 2017). KOK can protect transient cerebral global ischemia in gerbils. This is related to the attenuation of microglia activation and the increase of cytokine IL-1β (Cai et al., 2011). In the present study, KOK significantly prevented microglial activation and enhancement of mRNA expression levels of representative pro-inflammatory cytokines (IL-6, TNF-α), COX-2, and iNOS in the SNpc and striatum after MPTP intoxication (Figure 2). These beneficial effects might be supported by positive effects of components of KOK as follows. *P. ginseng* extract can exert anti-inflammatory role associated with inhibition of microglial activation in various *in vitro* and *in vivo* studies (Cho, 2012; Choi et al., 2015; Nabavi et al., 2015; Lee Y. M. et al., 2017). Compounds (specifically, seco-lanostane triterpenoid) from ethanol extract of sclerotia of *P. cocos* can inhibit iNOS and COX-2 protein expression levels in LPS-stimulated Raw264.7 cells (Lee S. R. et al., 2017). GYF-21, an epoxide 2-(2-phenethyl)-chromone derivative isolated from Chinese agarwood, can markedly inhibit the activation of microglia, dendritic cells, and neutrophils, all of which play important roles in innate immunity. Furthermore, KOK can significantly inhibit innate and adaptive immunity via suppressing STAT1/3 and NF-κB signaling pathways (Guo et al., 2017). Therefore, KOK might exert protective effect against MPTP neurotoxicity by inhibiting microglial activation and expression of inflammatory mediators based on its multiple materials and various targets.

The blood–brain barrier (BBB) makes restricted passage of various biological or chemical entities to brain tissues. BBB consists of astrocyte end-feet, pericytes, basal lamina, and endothelial cells with junctional complexes consisting of tight, gap, and adherens junctions (Abbott et al., 2006; Sandoval and Witt, 2008; Zlokovic, 2008). Disruption of the BBB by neurological disorders including PD or drugs can lead to impaired BBB function, thus compromising the CNS microenvironment (Abbott et al., 2006; Sandoval and Witt, 2008; Zlokovic, 2008). Recent studies have indicated that developing agents that can keep BBB integrity may be powerful preventive and therapeutic approaches for neurological diseases including PD (Abbott et al., 2006; Sandoval and Witt, 2008; Zlokovic, 2008). BBB disruption is related to alterations of astrocytes, junctional components in endothelial cells, and possibly pericytes (Abbott et al., 2006; Sandoval and Witt, 2008; Zlokovic, 2008). Many synthetic agents, natural agents (resveratrol and shikonin), and natural agents (Ginkgo biloba extract EGb761 and Korean red ginseng) can prevent BBB disruption by inhibiting astroglial activation and alteration of junctional components (Wan et al., 2014; Wang et al., 2014; Lee et al., 2016c; Zhao et al., 2017). In the present study, KOK interrupted astroglial activation and inhibited the increase in mRNA expression of adherens junctional molecules (ICAM-1 and VCAM-1) in the SNpc and striatum after MPTP intoxication. It also prevented the decrease of tight junctional molecules (ZO-1 and claudin-3) (Figure 3). Taken together, our results indicate that KOK may directly or indirectly show protective effect against MPTP-mediated neurotoxicity via preventing disruption of BBB integrity, although the exact underlying mechanism remains to be elucidated.

It has been implicated that MAPKs (ERK, JNK, and p38) and NF-κB signaling pathways are closely associated with pathophysiological processes including neuronal loss, neuroinflammation, and BBB disruption (Abbott et al., 2006; Sandoval and Witt, 2008; Zlokovic, 2008). Therefore, materials that can regulate both MAPKs and NF-κB signaling pathways with efficacy to prevent or cure PD via various mechanisms might be used as functional food or medications (Kim and Choi, 2010; Flood et al., 2011). In the present study, KOK treatment significantly blocked activation of ERK, JNK, and p38 as well as activation of NF-κB and IκBα in the SNpc and striatum after MPTP intoxication (Figure 4). These results can be indirectly explained by previous reports about components of KOK. For example, *P. ginseng* extract and its component gintonin can inhibit the activation of all subtypes of MAPKs and NF-κB pathways in SNpc and striatum of MPTP model (Choi et al., 2018a,b). The supercritical fluid extract of Lycium chinense Miller root can also inhibit melanogenesis in B16F10 cells by down-regulating both mitogen-activated protein kinases (MAPK) and protein kinase A (PKA) signaling pathways (Huang et al., 2014). Taken together, anti-inflammatory activity of KOK might have contributed to its protective effect against MPTP-induced neurotoxicity.

Oxidative stress due to reactive oxygen species may be a cause of a complex multifactorial PD (Riess and Krüger, 1999; Blesa et al., 2015). Nrf2 (a phase II antioxidant “master regulator”) stimulation and its nuclear translocation can attenuate dopaminergic neuronal death by parkinsonian neurotoxin such as MPTP and it active metabolite MPP^+^, rotenone, as well as hydrogen peroxide in *in vitro* and *in vivo* studies (Todorovic et al., 2016). In the present study, KOK upregulated expression levels of Nrf2 protein and representative Nrf2-dependent proteins HO-1 and NQO1 in the SNpc and striatum following MPTP intoxication (Figure 5). However, pre-inhibition of Nrf2 by Nrf2 inhibitor neutralized such protective effects (neurological behavior, expression of p38 MAPK and NF-κB, and nuclear translocation of Nrf2) of KOK on neurological dysfunction and neurotoxicity after MPTP intoxication (Figure 6). These results may be directly or indirectly explained by previous reports as follows. Microwave-assisted extract of *P. cocos* Wolf possesses antioxidant property *in vitro*, showing DPPH radical and hydroxyl radical reducing power and metal chelating ability (Wang et al., 2016). Ginsenosides and gintonin of *P. ginseng* can mitigate neurodegeneration by upregulating Nrf2-mediated antioxidant cascade both *in vitro* and *in vivo* (Cho, 2012; Nabavi et al., 2015; Lee Y. M. et al., 2017; Choi et al., 2018b). Collectively, these results indicate that KOK might be able to reduce anti-dopaminergic neuronal loss by its anti-oxidant activity.

## Conclusions

**Figure 7:**
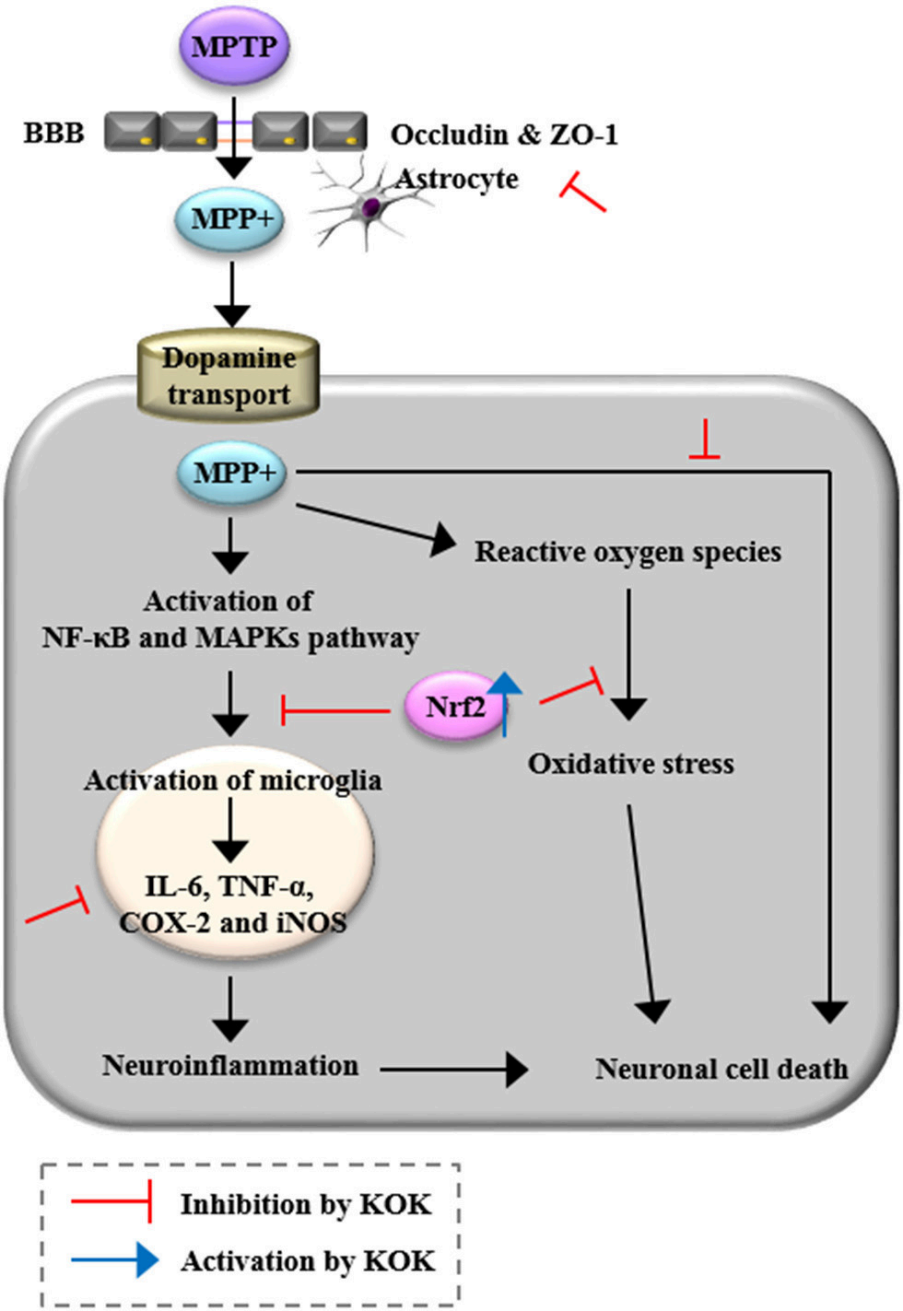
A diagram showing the proposed mechanisms of KOK in the SNpc and striatum following MPTP intoxication. KOK can protect neuronal death by MPTP intoxication via anti-neuronal death, anti-inflammatory, and anti-oxidative activities.

Current treatment for PD is unsatisfactory. One reason is that drivers of PD including neurotoxicity, microglial activity, BBB disruption, and oxidative stress are not simultaneously targeted. Developing a multifunctional therapy targeting these drivers of PD is very attractive but challenging. In the present study, we demonstrated that KOK, a traditional multi-herbal medicine, could markedly prevent neurodegeneration caused by MPTP-induced toxicity possibly by targeting multifactorial etiologies including anti-neuronal death, anti-inflammatory, and anti-oxidative activities (Figure 6). It could prevent BBB disruption by inhibiting MAPKs and NF-κB pathways while stimulating the Keap1-Nrf2 pathway (Figure 7). Taken together, our results suggest that KOK, a traditional multi-herbal medicine, may be useful as a functional food or medication to prevent and treat PD.

## Author Contributions

JHC performed the behavioral experiments, immunohistochemistry, and Western blots, and prepared all figures. MJ carried out real-time PCR assay and contributed to data interpretation. JIL cared animal and treated drugs. W-SC commented about KOK and contributed to data interpretation. I-HC conceived all experiments, analyzed the results, and wrote the manuscript. All authors have read and approved the final manuscript.

### Conflict of Interest Statement

The authors declare that the research was conducted in the absence of any commercial or financial relationships that could be construed as a potential conflict of interest.
